# Ligand Bias and Its Association With Pro-resolving Actions of Melanocortin Drugs

**DOI:** 10.3389/fphar.2018.00919

**Published:** 2018-08-14

**Authors:** Sara Patruno, Jose Garrido-Mesa, Mario Romano, Mauro Perretti, Trinidad Montero-Melendez

**Affiliations:** ^1^The William Harvey Research Institute, Barts and The London School of Medicine, Queen Mary University of London, London, United Kingdom; ^2^Department of Medical, Oral and Biotechnological Sciences, D’Annunzio University of Chieti-Pescara, Chieti, Italy; ^3^Center on Aging Sciences and Translational Medicine (CeSI-MeT), D’Annunzio University of Chieti-Pescara, Chieti, Italy; ^4^Centre for inflammation and Therapeutic Innovation, Queen Mary University of London, London, United Kingdom

**Keywords:** melanocortin, resolution pharmacology, inflammation, ligand bias, GPCR, functional selectivity

## Abstract

Resolution Pharmacology identifies drugs developed on the biology of the resolution phase of inflammation, the complex molecular and cellular network of events that ensure the tight temporal and spatial control on the inflammatory response. As such, new anti-inflammatory and pro-resolving drugs could derive from pro-resolving mediators and receptors. To implement faithful screening programs, however, it is important to rely on predictive signaling pathway relevant for the ultimate bio-action of interest. Herein we performed an analysis with four prototypical melanocortin receptor (MC_1,3,4,5_) agonists. The choice fell on the natural agonist αMSH, the small molecule BMS-470539, and the synthetic derivatives [D-Trp^8^]-γMSH and [Nle^4^,D-Phe^7^]-αMSH. We used human macrophages and quantified the effect of the four agonists on inhibition of cytokine release and promotion of efferocytosis. All agonists (1–10 μM) significantly inhibited cytokine release by LPS-stimulated cells whereas [D-Trp^8^]-γMSH was the most effective in inducing efferocytosis (∼60% increase). To study the signaling profile, we monitored cAMP accumulation and ERK1/2 phosphorylation, and constructed biased plots that revealed a marked biased profile of [D-Trp^8^]-γMSH toward phospho-ERK1/2. Correlation matrix analysis of all data pointed at phospho-ERK1/2 at any receptor as the most prominent pathway to attain pro-phagocytic actions, and MC_1_ receptor as the most relevant to drive anti-cytokine effects. In conclusion, the present study highlights the need to associate single-target signaling data with relevant functional outcomes. In this manner, we would increase our chances to optimize drug discovery programs during the early target validation and hit-to-lead phases.

## Introduction

The study of the *resolution* of inflammation as a specific feature of the whole inflammatory response was formally established one decade ago (Serhan et al., 2007), when the therapeutic potential of mimicking the way our own body terminates inflammation was envisaged. Since then, multiple pro-resolving molecular mediators have been discovered and the cellular mechanisms involved in the active termination of the inflammatory response defined (Ortega-Gomez et al., 2013). The field is now entering a new stage in which this new knowledge is being translated into novel drugs -many entering clinical development phase-, and drug discovery programs are being designed with the resolution approach in mind (Perretti et al., 2015). The melanocortin system (MC) constitutes one of such endogenous pro-resolving mechanisms, in addition to its multiple roles including energy homeostasis, skin pigmentation or steroidogenesis, thus presenting very diverse potential therapeutic applications (Leone et al., 2013; Montero-Melendez, 2015; Ferrante et al., 2016, 2017). However, the development of the “ideal" MC molecule is a proven challenge. Ideally, besides parameters such as potency or stability, an MC drug should also present receptor selectivity according to the intended indication. In addition, regarding the receptor activation profile induced by the drug, we still do not know what represents an ideal MC drug. For example, as cAMP activation is known to be essential for melanin production in melanocytes, it is unknown whether other functional outcomes (e.g., anti-inflammatory actions) require the same activation profile. The recent discovery of the existence of ligand bias at MC receptors (Buch et al., 2009; Montero-Melendez et al., 2015; Yang and Tao, 2017) suggests that in-depth studies on the relation between the signaling pathways activated by MCRs and functional outcomes should be conducted, as a better definition of this functional selectivity may lead to better drugs with improved safety and efficacy profiles.

In this *Brief Report* we used human monocyte-derived macrophages and present an association analysis between melanocortin functional outcomes and signaling pathways engaged by the four prototypical agonists, widely used compounds in melanocortin research. We propose that this systematic approach can reveal new opportunities for drug discovery.

## Materials and Methods

### Chemical Compounds

The following drugs were used: αMSH, BMS-470539 (BMS), [Nle^4^,D-Phe7]-αMSH (NDP), and [D-Trp^8^]-γMSH (DTrp) (Tocris); M-CSF (PeproTech); LPS *E. coli* O111:B4 (Sigma).

### Isolation of Human Primary Cells

Experiments using healthy volunteers (written consent provided) were approved by P/00/029 East London and The City Local Research Ethics Committee 1. Blood was collected into 3.2% sodium citrate, diluted 1:1 with RPMI-1640 and separated through a double-density gradient using Histopaque 1077/1119 (Sigma-Aldrich). PBMCs were collected from the top layer and differentiated into macrophages in complete media + 50 ng/ml M-CSF during 7 days. Neutrophils were collected from the middle layer and incubated in 10% FCS overnight at 37°C, 5% CO_2_ to let neutrophils undergo spontaneous apoptosis.

### Stimulation of Differentiated Primary Macrophages

Differentiated macrophages were stimulated with 1 ng/ml LPS, 30 min post drug treatments. Supernatants were collected 18 h later and analyzed by ELISA. Melanocortin drugs concentrations were chosen according to previous reports (Montero-Melendez et al., 2011).

### Cell Transfections

HEK293A cells were maintained in DMEM containing 10% FCS and 1% penicillin/streptomycin and kept at 37°C with 5% CO_2_. Cells were transfected with *MC1R*, *MC3R*, *MC4R* or *MC5R* TrueORF cDNA clones (Origene) using Lipofectamine 2000 (Invitrogen) and OptiMEM according to manufacturer’s instructions and used after 24 h.

### Gene Expression

Ribonucleic acid (RNA) was extracted using PureLink RNA Mini Kit with DNase I digestion (Thermo Scientific). cDNA was synthesized (1 μg RNA) with SuperScript VILO MasterMix (Invitrogen). End-point PCR was performed with ReddyMix PCR Master Mix (Thermo Scientific) and amplified products visualized by 3% agarose electrophoresis. To account for genomic DNA contamination, negative cDNA controls (i.e., without reverse transcriptase) were used. Quantitect primers (QIAGEN/amplicon size) used are the following: *MC1R* (QT01004241/137bp), *MC2R* (QT01155007/118bp), *MC3R* (QT00209895/74bp), *MC4R* (QT00245595/89bp), *MC5R* (QT00211960/146bp), *POMC* (QT00001204/126bp), *PCSK1* (QT00013853/139bp), *PCSK2* (QT00054754/126bp), *MRAP* (QT00103866/86bp), *MRAP2* (QT00493150/113bp), *GAPDH* (QT00079247/95bp), and *HPRT1* (QT00059066/130bp).

### ELISA and EIA Assays

The following kits were used following manufacturer’s instructions: cAMP Select EIA kit (Cayman Chemical); ERK1/2 (pT202/Y204) SimpleStep ELISA Kit (Abcam); CCL-2, IL-6, IL-10, and IL-8 Ready-SET-Go ELISA (eBioscience).

### Efferocytosis Assay

Differentiated macrophages were stimulated with compounds/vehicle for 30 min before the addition of apoptotic neutrophils (1:5 macrophage to neutrophil ratio) for 1 h. Cells were fixed and stained using the myeloperoxidase (MPO) assay by adding 0.1 mg/ml of dimethoxybenzidine (Sigma-Aldrich) and 0.03% (v/v) hydrogen peroxide for 1 h. Cells were analyzed by light microscopy with three random fields being acquired per well. Clearance Index: (%Phagocytosis × %Multiple ingestions)/100.

### Statistical Analysis

Statistical parameters including the exact value of n for each experiment, nature of data shown (mean ± SE) and statistical significance are reported in Figure Legends. Data is judged to be statistically significant when *p* < 0.05. Statistical analysis was performed in GraphPad PRISM v7.

## Results

### Melanocortin Pathway Expression in Primary Human Macrophages

**FIGURE 1:**
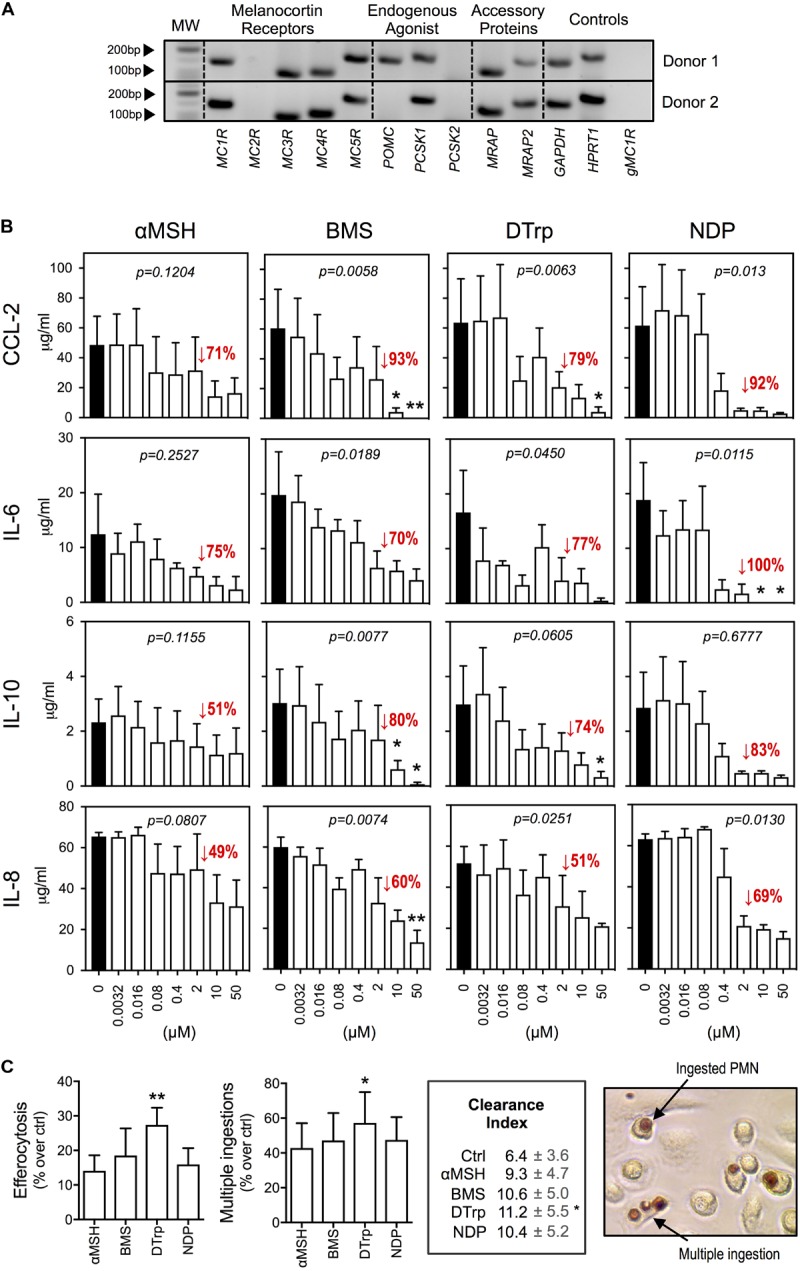
Effects of melanocortin drugs on macrophage function. **(A)** Expression of members of the MC pathway by PCR. Two representative donors are shown. Lack of amplification from genomic DNA is reported in the last line. **(B)** Macrophages were stimulated with LPS (1 ng/ml) 30 min after the addition of melanocortin drugs at the indicated concentrations. Supernatants were collected after 18 h and cytokines measured by specific ELISA. **(C)** Macrophages were treated with the indicated melanocortin drugs (10 μM) for 30 min prior to the addition of apoptotic neutrophils. The MPO assay was performed 1 h later to specifically stain ingested neutrophils. Phagocytosis was calculated as % of macrophages containing at least one neutrophil inside. Multiple ingestions were quantified as % of phagocytic macrophages that ingested more than 1 neutrophil. Clearance Index = (%phagocytosis × %multiple ingestions)/100. Values for basal (ctrl) phagocytosis and multiple ingestions were 27 and 15%, respectively. Photograph shows the dark brown coloration selectively acquired by neutrophils after performing the MPO assay. Data represent mean ± SEM of 3 independent human donors and were analyzed by repeated measures one-way ANOVA (*p* values shown in graphs) followed by Dunn’s multiple comparison test (^∗^*p* < 0.05, ^∗∗^*p* < 0.01).

Expression profile analyses by qPCR (**Figure 1A**) indicated presence of the gene products for the receptors *MC1R*, *MC3R*, *MC4R*, and *MC5R.* The processing enzyme *PCSK1* and the accessory proteins melanocortin-2 receptor accessory protein 1 and 2 (*MRAP*, *MRAP2*) were also expressed. The expression of *POMC* gene was donor-dependent as it was detected in 25% of donors tested (*n* = 4). Taken together, human macrophages express multiple members of the MC pathway.

### Melanocortin Agonists Reduce LPS-Induced Macrophage Activation

The ability of melanocortin ligands to reduce cytokine release by macrophages challenged with inflammatory stimuli was studied using: αMSH (natural peptide, pan-agonist), BMS-470539 (*BMS*, synthetic small molecule, MC_1_ selective), [D-Trp^8^]-γMSH (*DTrp*, synthetic peptide, preference for MC_3_ over the other receptors) and [Nle^4^,D-Phe^7^]-αMSH (*NDP*, synthetic stable peptide, pan-agonist). LPS stimulation induced a marked increase in the release of CCL-2, IL-6, IL-10, and IL-8. All compounds reduced LPS-dependent cytokine release in a concentration-dependent manner (**Figure 1B**). Efficacy was lower for the natural agonist αMSH compared to the synthetic ones. The highest efficacy was observed for NDP achieving complete abrogation of IL-6 release at 10 μM.

### Pro-resolving Actions of Melanocortin Agonists

We next investigated the pro-resolving actions of these molecules by assessing promotion of efferocytosis, a key resolution mechanism that promotes the non-phlogistic clearance of dead cells after the inflammatory acute phase. All compounds augmented efferocytosis of human apoptotic neutrophils by primary macrophages (statistically significant for DTrp), as compared with non-treated (ctrl) cells (**Figure 1C**). We also quantified multiple ingestions, as they reflect the effectiveness of dead cells clearance (i.e., whether they also “eat” more). DTrp induced a 57% increase in the number of macrophages performing multiple ingestions. Together, the clearance index showed increased effectiveness in apoptotic cell clearance for all compounds.

### Existence of Ligand Bias on Melanocortin Receptor Activation

cAMP activation by melanocortin drugs has been extensively studied while ERK1/2 activation is much less explored. We confirm the MC drugs used in this report can induce ERK1/2 phosphorylation in primary human macrophages (∼15–40% increase at 2 μM after 5 min stimulation – data not shown).

**FIGURE 2:**
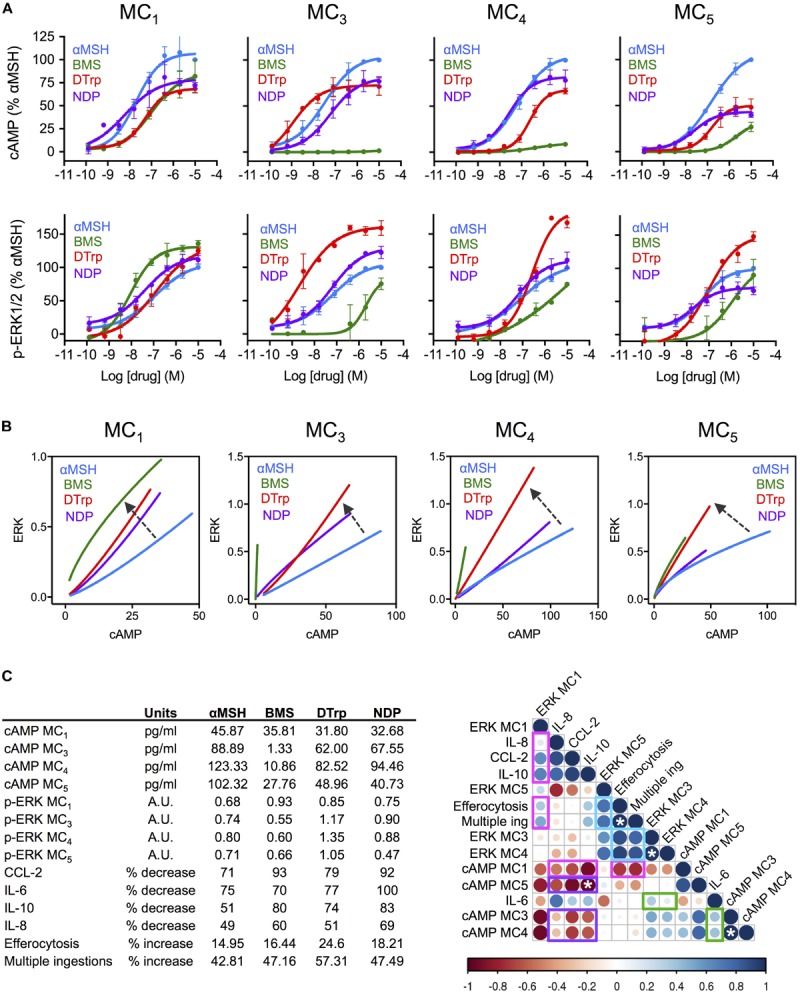
Association between signaling cascades and functional effects of melanocortin drugs on macrophages. **(A)** Concentration dependent accumulation of cAMP and formation of ERK1/2 phosphorylation (p-ERK1/2) upon melanocortin stimulation of transiently transfected cells. Data represent mean ± SEM of *n* = 2–3 independent experiments, each one in duplicate. **(B)** Bias plots for each MC receptor were constructed using equimolar concentrations (using full response curves 10 μM–0.13 nM) at the two pathways cAMP (pg/ml) and p-ERK1/2 (arbitrary units, AU), presenting one variable as a function of the other. Arrows indicate biased signaling respect to the natural agonist αMSH. **(C)** A correlation analysis was performed using all data generated in the present study using values for 10 μM: cAMP (pg/ml) and ERK1/2 phosphorylation (AU), reduction of cytokines release (%) and increase on phagocytosis and multiple ingestions (%), all shown in the insert. The analysis was performed by Pearson correlation test. Strength of the association is denoted by both color and size of bubble (darker color and bigger size meaning higher correlation; red, negative; blue positive). Bar indicates correlation coefficient “*r*” ranging from *r* = –1 (negative correlation) to *r* = 1 (positive correlation). White asterisks indicate statistically significant association (^∗^*p* < 0.05).

In our next approach, we linked biological properties to cell signaling, generating concentration response curves for both cAMP accumulation and ERK1/2 phosphorylation using HEK-293 cells transfected with each single receptor under investigation (**Figure 2A**). Bias plots were then constructed by representing one pathway against the other (**Figure 2B**). Ligand bias was analyzed in reference to the endogenous αMSH, i.e., considering the response induced by αMSH as the reference, and as such any deviation from that would indicate existence of biased signaling. Interestingly, DTrp presented preference for ERK1/2 phosphorylation over cAMP engagement at all the MCRs. BMS showed a similar trend, with a clear shift toward phospho-ERK1/2 compared to αMSH on MC_1_. On the other receptors, the preference of BMS for phospho-ERK1/2 was more evident given its partial agonistic activity (although with very low potency) on MC_3-5,_ while no significant activity was attained on the cAMP pathway. This effect of BMS (reported as an MC_1_-selective drug) is of particular relevance, as it highlights the importance of measuring signaling outcomes other than the canonical cAMP to confidently attribute receptor selectivity to a molecule. To a lesser extent, the αMSH derived peptide NDP also presents some degree of ligand bias toward phospho-ERK1/2 at MC_1_ and MC_3_.

### Association of Melanocortin Distinct Signaling Profiles With Functional Outcomes

To determine whether bias signaling patterns lead to functional consequences we generated a correlation matrix compiling all the data produced in this study at 10 μM: efficacy at either signaling pathway, cytokine reduction and increase in efferocytosis. We could then highlight several associations that may help elucidate the contribution of each receptor type and signaling pathways to functional outcomes (**Figure 2C**). For example, the only receptor positively associated with both reduction in cytokines and increased phagocytosis was MC_1_ (highlighted in magenta). Moreover, this positive association was only found with phospho-ERK1/2-MC_1_, while the association with cAMP-MC_1_ was indeed the opposite. Strikingly, cAMP pathway at all receptors was negatively associated with cytokine reduction (highlighted in purple). The case of IL-6 showed, however, a different trend, with positive (although weak) association with phospho-ERK1/2 and cAMP at both MC_3_ and MC_4_ (highlighted in green). The analysis of phagocytosis also revealed new clues about the relevant signaling that may drive this effect. In addition to phospho-ERK1/2 at MC_1_, as mentioned earlier, ERK1/2 phosphorylation at all other receptors was also positively and strongly associated with increase in efferocytosis and multiple ingestions (highlighted in blue). This is in agreement with the high pro-phagocytic effect obtained with DTrp shown previously on **Figure 1C**, and the biased signaling that this receptor presents toward phospho-ERK1/2, as shown in **Figure 2B**.

## Discussion

G-protein coupled receptor (GPCRs) activation is a highly dynamic process where receptor proteins can acquire multiple active states evoking distinct signaling pathways (Kroeze et al., 2003). The complex pharmacology of these receptors is determined by properties like ligand promiscuity, temporal pathways network activation, desensitization, ligand independent activation, allosteric modulation or ligand bias (Melancon et al., 2012; Kenakin and Christopoulos, 2013; Montero-Melendez et al., 2017; Sexton and Christopoulos, 2018), presenting an arduous challenge, yet a unique opportunity, for innovative drug discovery. The exploitation of the full potential of these receptors, like MCRs, requires a better understanding of their behavior and discarding the old view of GPCRs as a plain linear sequence of events of “ligand-receptor-pathway-effect.”

Here we performed a systematic analysis selecting two major biological actions of MCR agonists and studied their association (positive or negative) with two signaling readouts. Since we used four distinct prototypical agonists at these receptors (all of them expressed in human macrophages), combining natural with synthetic compounds, peptides with small molecules, the study is in itself novel and may pave the way to a “bio-matrix” based approach relevant for drug discovery programs. In fact, our findings represent a challenge against the current dogma on cAMP drug discovery approaches, assuming cAMP as the major, or even the only pathway used to inform candidate selection. Surprisingly, cAMP pathway at all receptors was markedly negatively associated with the ability to reduce cytokine release, while ERK1/2 phosphorylation at MC_1_ and MC_3,4,5_ were positively associated with desirable effects on cytokine release and promotion of phagocytosis, suggesting a mayor role for this non-canonical pathway in the pro-resolving and anti-inflammatory actions of MC drugs. This confirms our recent discovery of an ERK1/2-biased melanocortin small molecule (AP1189) that presents anti-inflammatory actions despite no induction of cAMP (Montero-Melendez et al., 2015). In fact, the ERK1/2 inhibitor FR180204 completely abrogated the pro-efferocytic effect of AP1189. Currently, all drug screening programs on MC drug discovery are based on cAMP accumulation, despite the lack of consistent evidence that this pathway is indeed the most therapeutically relevant one, at least to develop new anti-inflammatory agents. Thus, this promising candidate (currently on phase I trial–CT#2016-004171-48-) would have been filtered out in a typical cAMP-based screening. This analysis also revealed interesting differences between cytokine reduction and promotion of phagocytosis, where the latter seems to be strongly dependent on ERK1/2 phosphorylation at MC_3,4,5_ rather than MC_1_.

Correlation analyses need to be interpreted considering that association does not imply causation. Therefore, more in-depth analyses are necessary to fully understand associations between receptors-pathways-functions to inform drug discovery programs to prevent decisions based on assumptions. Here, we propose that a “bio-matrix” based approach would enable a better compound profiling, facilitating candidate selection for follow up development while, at the same time, ensuring the relevant biological properties are taken into consideration early-on to inform this selection. Furthermore, macrophages express multiple melanocortin receptors which are activated simultaneously by a given agonist. However, drug discovery screenings are usually performed on a single target basis using transfected cell as we did here. Our study then highlights the relevance of understanding single-target signaling data and its association with “real-cells” functional outcomes to better define the relevant parameters to optimize drug discovery programs during early target validation and hit-to-lead phases.

## Author Contributions

TM-M conceived the study. SP and JG-M contributed to the experimental methods. TM-M, SP, and JG-M analyzed the data. TM-M, MP, SP, and JG-M interpreted the data. TM-M contributed to the visualization. TM-M and MP wrote the manuscript. SP, JG-M, and MR reviewed the manuscript. MP, TM-M, and MR acquired funding. TM-M supervised the study.

## Conflict of Interest Statement

The authors declare that the research was conducted in the absence of any commercial or financial relationships that could be construed as a potential conflict of interest. The reviewer GO declared a shared affiliation, though no other collaboration, with two of the authors MR and SP.
